# Tumefactive Multiple Sclerosis: Challenges With Treatment Modalities

**DOI:** 10.7759/cureus.41845

**Published:** 2023-07-13

**Authors:** Jude ElSaygh, Nicole Kandinova, Anas Zaher, Gurinder K Sunner, Sofya Kostanyan

**Affiliations:** 1 Internal Medicine, NewYork Presbyterian - Brooklyn Methodist Hospital, New York, USA

**Keywords:** high-dose corticosteroids, multiple sclerosis flare-up, intravenous immunoglobulins (ivig), tumefactive sclerosis, diagnosis of multiple sclerosis

## Abstract

Tumefactive multiple sclerosis comprises a rare subset of multiple sclerosis that often poses a diagnostic challenge to physicians. It is unique in its presentation as a solitary lesion, usually larger than 2 cm, with surrounding vasogenic edema, commonly mimicking a primary intracranial malignancy. We present a case of a 25-year-old female with no significant past medical history who presented to our institution with homonymous superior quadrantanopia. During her admission, she underwent a magnetic resonance imaging (MRI) of the brain, which revealed a large lesion in the left temporal area surrounded by marked edema. A thorough workup revealed a diagnosis of tumefactive multiple sclerosis. Subsequently, she was initiated on intravenous immunoglobulin rather than stress dose steroids, given the concern for a superimposed infection. Interestingly, the patient had a paradoxical progression of her symptoms as well as expansion of the vasogenic edema on a repeat MRI. In our case, we highlight the key differences in tumefactive multiple sclerosis diagnosis and management.

## Introduction

Tumefactive multiple sclerosis (TMS) represents a rare form of multiple sclerosis characterized by a solitary lesion usually larger than 2 cm with surrounding edema. Although its incidence is thought to be 3 out of a million people per year, its recognition is nonetheless important [1]. In contrast to the multiple and small periventricular lesions most commonly seen in multiple sclerosis, the space-occupying nature of TMS can be mistaken for a primary CNS tumor. This common misconception can lead to delays in the diagnosis and treatment of this autoimmune condition. Our case describes the clinical course of a young female who presented to our institution with homonymous superior quadrantanopia. We highlight key findings in our experience to clue the reader to recognizing TMS. In addition, we found a peculiar reaction to intravenous immunoglobulin (IVIG), which led to paradoxical worsening of our patient's symptoms.

## Case presentation

**Figure 1:**
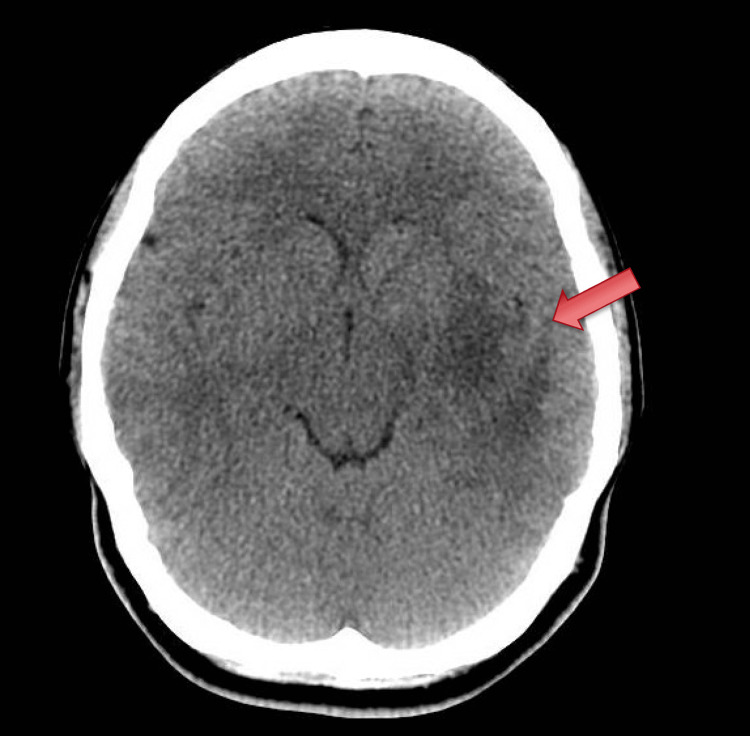
Non-contrast CT of the head revealing a large area of vasogenic edema in the left frontal and temporal lobes. CT, computed tomography

**Figure 2:**
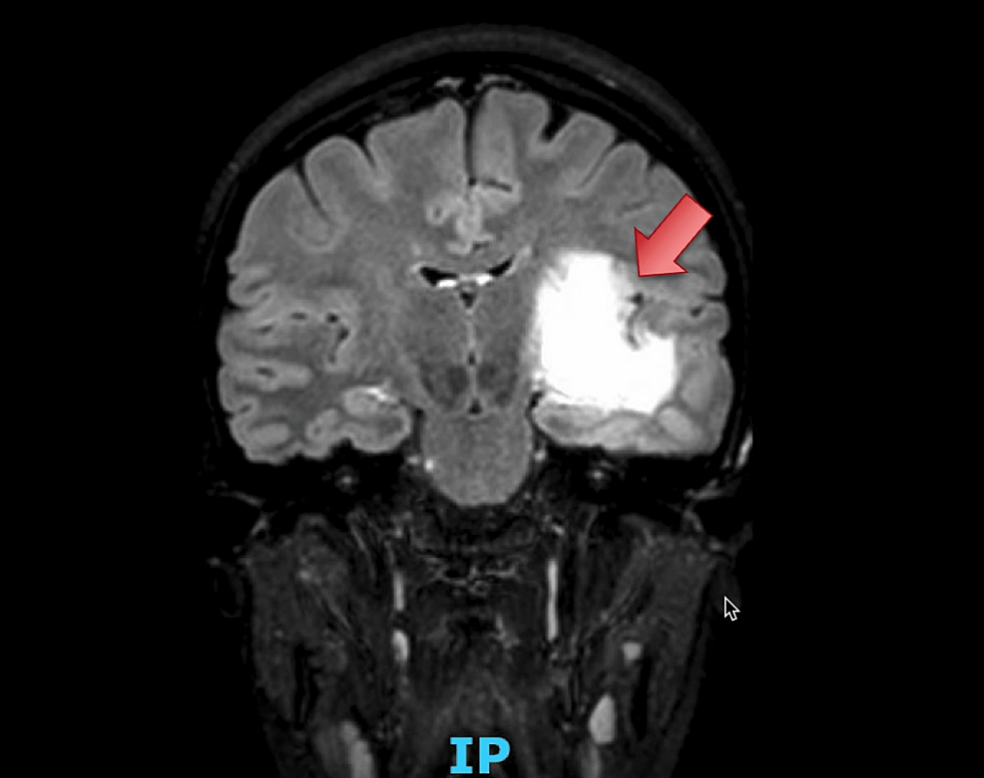
T2 FLAIR frontal sections of brain MRI showing an expansile lesion with surrounding vasogenic edema involving the left temporal stem, posterior subinsular region, posterior basal ganglia, and posterior limb of the internal capsule. FLAIR, fluid-attenuated inversion recovery; MRI, magnetic resonance imaging

A 25-year-old female without a significant past medical history was referred by her ophthalmologist for new-onset homonymous superior quadrantanopia. Upon arrival at the emergency room, a non-contrast CT of the head revealed a large area of vasogenic edema in the left frontal and temporal lobes, as shown in Figure 1. A subsequent magnetic resonance imaging (MRI) confirmed the presence of an expansile lesion with surrounding vasogenic edema involving the left temporal stem, posterior subinsular region, posterior basal ganglia, and posterior limb of the internal capsule, as shown in Figure 2. Despite initial concern for malignancy, a lumbar puncture was performed to evaluate possible infectious, paraneoplastic, or demyelinating processes. Cerebrospinal fluid obtained resulted positive for oligoclonal bands and myelin basic protein. Amidst the patient's visual deficits, she was also notably tachycardic throughout her hospital stay and had an isolated episode of fever.

**Figure 3:**
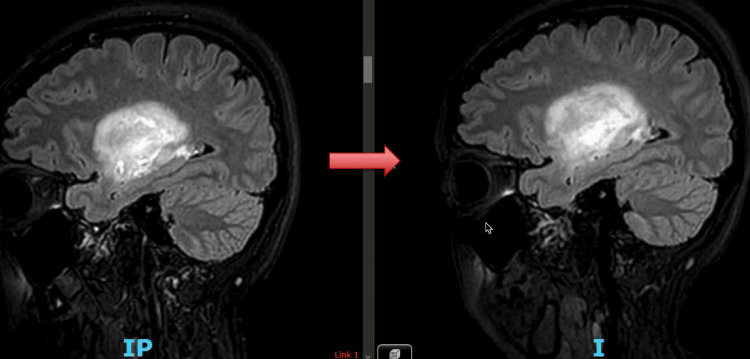
T2 FLAIR sagittal section of brain MRI showing extension of vasogenic edema on the right image after IVIG administration. FLAIR, fluid-attenuated inversion recovery; MRI, magnetic resonance imaging; IVIG, intravenous Immunoglobulin

Further evaluation revealed a diagnosis of Graves' disease, which raised our suspicion for a concomitant autoimmune diagnosis of TMS. The decision was made to begin treatment with IVIG rather than with stress steroids due to concern for possible underlying infection. Interestingly, the patient's initial complaint of homonymous superior quadrantanopia soon progressed to homonymous hemianopia. This was accompanied by worsening right-sided numbness and tingling. A repeat MRI obtained five days after initiation of IVIG revealed extension of the vasogenic edema, as shown in Figure 3. The patient was subsequently started on high-dose corticosteroids. After a three-day course of 100 mg of daily IV methylprednisolone, she reported significant improvement in her vision and sensory deficits. She was eventually discharged with instructions for close outpatient neurology follow-up.

## Discussion

TMS is a rare subset of multiple sclerosis. The prevalence of TMS is estimated to be 1-3/1,000 cases of multiple sclerosis or three cases per million per year in the general population. TMS may occur at any age but is most often seen in ages 20-30 [1], with roughly 75% of patients being female. To this day, the pathogenesis of TMS remains unknown. In a large multicenter retrospective study evaluating patients with TMS, 10% of patients had comorbid autoimmune diseases [2]. As in our case, the patient's new diagnosis of Graves' disease further clued us into the diagnosis of TMS. The typical presentation of TMS includes a large intracranial lesion measuring greater than 2.0 cm with noticeable perilesional edema. TMS most commonly affects the frontal and/or temporal lobes. The lesion may present as a wide array of symptoms depending on its location [3]. Motor, sensory, cognitive, and cerebellar symptoms are predominant. The marked edema may lead to symptoms of increased intracranial pressure such as headaches, and, at times, cerebral herniation requiring prompt management [1]. The diagnostic workup of TMS should include brain and spinal cord MRIs to help distinguish the lesion from a brain neoplasm. Incomplete ring enhancement, a higher number of lesions, and the absence of mass effect are more characteristic of TMS. A lumbar puncture may reveal oligoclonal protein bands and myelin basic protein in up to 74% of patients. Brain biopsies are reserved for diagnostically challenging patients in whom conservative diagnosis and management fail [4].

High-dose corticosteroids are considered the first-line treatment in the subset of patients with TMS. Approximately 86% of patients show improvement with treatment. In those patients without symptomatic improvement, plasmapheresis is considered as second-line therapy [5]. IVIG has been shown to be beneficial in decreasing the number of relapses and improving disability in patients with multiple sclerosis [6]. However, this has not been explicitly studied in the treatment of TMS. In our review of the English literature, our case seems to be the first to describe worsening of TMS with IVIG. This finding warrants more research into the effects of IVIG in TMS. Cyclophosphamide has been referenced as potentially effective in improving clinical and radiological disease activity in those cases of TMS that are refractory to corticosteroids and plasmapheresis [7]. The role of disease-modifying agents in patients with TMS is unclear. Interferon-beta and glatiramer acetate are preferred. However, these agents are usually not initiated until a second clinical attack. Fingolimod and natalizumab have been noted to be associated with several cases of increasing tumefactive demyelination and thus should be avoided in patients with TMS. It is interesting to note that increased tumefaction with fingolimod, natalizumab, and, in our case, IVIG suggests that TMS can behave very differently than traditional multiple sclerosis. These differences in its treatment are important to consider when approaching patients with TMS.

The long-term prognosis of TMS is variable. Approximately one-third of patients will not develop a subsequent demyelination attack. In the subset of patients who do, two-thirds of them will follow the relapsing-remitting course. Early recognition of this rare disease and its appropriate management are paramount in improving outcomes in patients diagnosed with TMS.

## Conclusions

TMS remains a challenging disease in terms of its pathogenesis and diagnosis. Despite being categorized as a subset of multiple sclerosis, our experience has taught us that it can behave differently than the traditional multiple sclerosis we commonly see. Increased tumefaction with IVIG therapy in our case highlights the need for further research in the field of this often misdiagnosed phenomenon.
